# Interventions to prevent postoperative atrial fibrillation in Dutch cardiothoracic centres: a survey study

**DOI:** 10.1007/s12471-023-01849-1

**Published:** 2024-02-15

**Authors:** Angelique Emiola, Jolanda Kluin, Sulayman el Mathari, Joris R. de Groot, Wim-Jan van Boven, the Cardiothoracic Surgery Data Registration Committee of the Netherlands Heart Registry

**Affiliations:** 1 Department of Cardiothoracic Surgery, Amsterdam University Medical Centres, Amsterdam, The Netherlands; 2 Department of Cardiothoracic Surgery, Erasmus Medical Centre, Rotterdam, The Netherlands; 3 Department of Experimental and Clinical Cardiology, Amsterdam University Medical Centres, Amsterdam, The Netherlands

**Keywords:** Atrial fibrillation, Postoperative complications, Surveys, Questionnaires

## Abstract

**Introduction:**

Postoperative atrial fibrillation (POAF) is a common phenomenon following cardiac surgery. In this study, we assessed current preventive strategies used by Dutch cardiothoracic centres, identified common views on this matter and related these to international guidelines.

**Methods:**

We developed an online questionnaire and sent it to all cardiothoracic surgery centres in the Netherlands. The questionnaire concerned the management of POAF and the use of pharmaceutical therapies (beta-blockers and calcium antagonists) and non-pharmaceutical methods (posterior left pericardiotomy, pericardial flushing and epicardial botulinum toxin type A injections). Usage of electrical cardioversions, anticoagulants and left atrial appendage closure were also enquired.

**Results:**

Of the 15 centres, 14 (93%) responded to the survey and 13 reported a POAF incidence, ranging from 20 to 30%. Of these 14 centres, 6 prescribed preoperative AF prophylaxis to their patients, of which non-sotalol beta-blockers were prescribed most commonly (57%). Postoperative medication was administered by all centres and included non-sotalol beta-blockers (38%), sotalol (24%), digoxin (14%), calcium antagonists (13%) and amiodarone (10%). Only 2 centres used posterior left pericardiotomy or pericardial flushing as surgical manoeuvres to prevent POAF. Moreover, respondents expressed the need for guidance on anticoagulant use.

**Conclusion:**

Despite the use of various preventive strategies, the reported incidence of POAF was similar in Dutch cardiothoracic centres. This study highlights limited use of prophylactic amiodarone and colchicine, despite recommendations by numerous guidelines, and restricted implementation of surgical strategies to prevent POAF.

**Supplementary Information:**

The online version of this article (10.1007/s12471-023-01849-1) contains supplementary material, which is available to authorized users.

## What’s new?

- This survey showed that most Dutch cardiothoracic centres did not prescribe preoperative prophylaxis for postoperative atrial fibrillation (POAF) despite positive recommendations.
- Specifically, colchicine and amiodarone were not implemented as prophylaxis, despite strong recommendations by multiple guidelines.
- Usage of non-pharmaceutical intervention methods and predictive risk scores were limited among Dutch cardiothoracic centres.
- Most respondents expressed the need for uniform management of POAF and better guidelines on anticoagulant prescription (specifically duration).

## Introduction

Postoperative atrial fibrillation (POAF) is frequently seen after cardiac surgery and is associated with heart failure, longer hospitalisation, stroke and increased mortality [1, 2]. In this respect, POAF could serve as a relevant discriminative marker for future cardiovascular risks [3]. Early POAF is defined as new-onset AF that occurs within the 30-day postoperative period and usually has a self-limiting course over 5–7 days. Local management protocols consist of treatment strategies once patients present with POAF. However, there is an increasing interest in ways to prevent this complication altogether instead of merely treating it. Still, the debate remains whether one should aim to eliminate POAF completely, considering POAF could serve as a potential marker for atrial myocardiopathy rather than a cause of detrimental outcomes.

According to the European Society of Cardiology (ESC) in collaboration with the European Association for Cardio-Thoracic Surgery (EACTS), the incidence of AF after cardiac surgery is 15–45% [4]. To prevent POAF, the ESC/EACTS Guidelines favour the use of perioperative beta-blockers and/or amiodarone and correction of electrolyte imbalances. Posterior left pericardiotomy and bi-atrial pacing are also recommended. The Canadian Cardiovascular Society recommends a ventricular response rate- or rhythm-control strategy [5]. Prophylactic sotalol or amiodarone were suggested in case other beta-blockers are contra-indicated [6]. Colchicine is the only anti-inflammatory agent recommended to prevent POAF. Other recommended preventive agents are ranolazine and digoxin [7–9].

Non-pharmacological preventative measures include posterior left pericardiotomy, which entails cutting the posterior pericardium. This allows drainage of excess blood and fluid, prevents inflammation and reduces the tendency of AF to occur [10–12]. Alternative methods are active tube clearance to minimise common chest tube occlusion [13], and perioperative pericardial flushing by continuously rinsing the pericardial space with irrigation solution [14]. Botulinum toxin type A (BoNT/A), injected into the atrial or epicardial fat pad, causes temporary neuromodulation and is associated with fewer occurrences of POAF [15]. Left atrial appendage closure (LAAC) is a procedure that does not prevent AF but could reduce thromboembolic events [16]. Although studies have shown LAAC may actually increase the risk of POAF [17, 18], the LAAOS III trial demonstrated it prevented stroke among patients with AF, both in the presence and absence of anticoagulation [19].

The aim of this study was to examine the ways preventive strategies for POAF are implemented in Dutch cardiothoracic centres and to evaluate their effectiveness. Usage of predictive risk scores were examined, e.g. the POAF risk score, which was developed to predict the probability of POAF [20]. Application of LAAC was also enquired.

## Methods

An online survey was developed for all 15 Dutch cardiothoracic centres. The questionnaire comprised 27 open-ended and closed questions about preventive strategies for POAF in patients undergoing on-pump sternotomy.

We assessed the POAF incidence, anticoagulant strategy, perceived complications and usage of the POAF risk score and different tools for POAF prophylaxis among centres. These tools included pharmaceutical and non-pharmaceutical interventions (posterior left pericardiotomy, perioperative pericardial flushing and BoNT/A injections). Respondents were asked about LAAC and electrical cardioversion (ECV) use and whether they had suggestions for qualitative improvement.

The surveys were sent via e‑mail to the local Cardiothoracic Surgery Registration Committee members of all Dutch heart centres. Non-responders were sent reminders. Data were stored in a password-protected environment.

## Results

### Incidence of postoperative atrial fibrillation

**Fig. 1:**
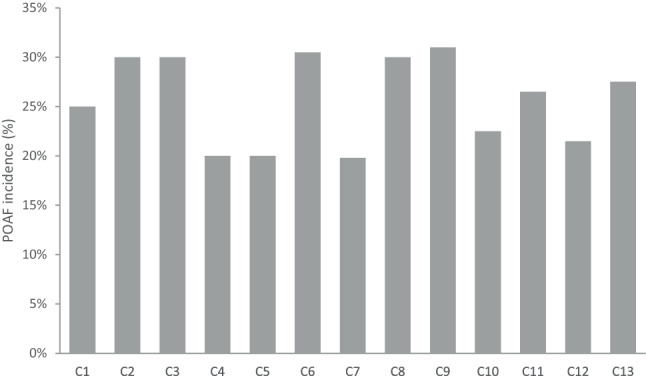
Reported incidence of postoperative atrial fibrillation (*POAF*) after cardiac surgery in 13 Dutch cardiothoracic centres

Of the 15 Dutch cardiothoracic centres, 14 (93%) responded to the survey, but not all centres provided an answer to each question. Completion rates per question are outlined in Table S1 in the Electronic Supplementary Material. Thirteen centres reported a POAF incidence, ranging from 20 to 30% (median 27%). The reported incidences per centre are presented in Fig. 1.

### Preoperative prophylaxis

Nine of the 14 centres (64%) used local protocols for POAF prophylaxis and treatment, consisting of administration of prophylactic beta-blockers and an anticoagulant regimen once POAF occurred. Two centres specified the prescribed dose of beta-blockers (i.e. 25 mg twice daily or 80 mg once daily). One centre also described perioperative electrolyte regulation in its protocol. One centre followed European guidelines instead of a protocol.

**Fig. 2:**
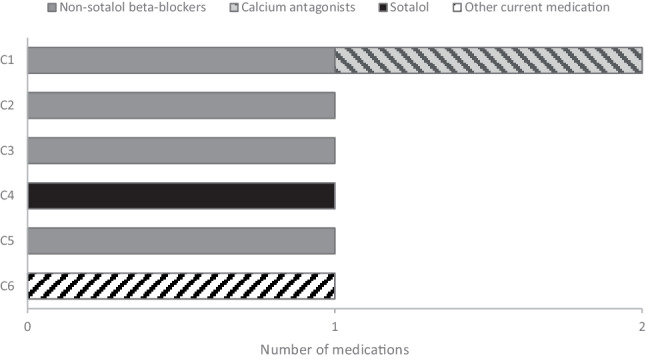
Reported prescription of preoperative prophylactic medication for atrial fibrillation in Dutch cardiothoracic centres

Six centres (43%) prescribed pharmaceutical prophylaxis preoperatively. This included non-sotalol beta-blockers (4/6; 67%), calcium antagonists (1/6; 17%), sotalol (1/6; 17%) and other current medication (1/6; 17%). Three centres prescribed prophylaxis to all patients, 2 centres prescribed prophylaxis only to patients who were already on beta-blockers or calcium antagonists, and 1 centre prescribed an anti-arrhythmic drug to patients already being treated with the specific drug. Colchicine was not used at all. The reported prescription of preoperative medications for AF prophylaxis in 6 centres is outlined in Fig. 2.

### Non-pharmaceutical prevention

Of the respondents, 12 (86%) believed posterior left pericardiotomy can prevent POAF, but only 1 centre used this intervention. Two centres (14%) were positive about this technique. The major reason for non-use was a lack of positive evidence (11/14; 79%). Similarly, most centres (11/14; 79%) believed perioperative pericardial flushing potentially reduces POAF, but only 1 centre used it in a research setting and none of them in a clinical context. All 11 centres found the evidence to be insufficient, and 2 centres (14%) stated there were no perceived benefits. One centre was positive about its effectiveness. Eight centres (57%) were familiar with BoNT/A injections in the atrial fat pad, but none used it to prevent POAF due to a lack of scientific evidence.

### Left atrial appendage closure

Most centres (10/14; 71%) routinely performed concomitant LAAC for patients with pre-existent AF. Of them, 30% was aware this entailed procedure may increase the risk of POAF. Of the remaining 4 centres not routinely performing LAAC, 3 were not aware of this risk and 1 centre was. Indications to perform LAAC varied. Eight of the 14 centres (57%) expected to alter current indications in response to the LAAOS III trial results [19]. Two centres (14%) performed LAAC exclusively in surgical ablation, but one of them seldomly performed LAAC for patients with chronic AF, 3 centres reserved LAAC for patients with pre-existent AF undergoing open-heart surgery, and 1 centre did not state an indication.

### Postoperative prophylaxis and treatment

All 14 centres administered postoperative medication. Their first line of medication was standard AF prophylaxis, while the second and subsequent lines of medication were administered in the clinical phase. The majority (12/14; 86%) prescribed non-sotalol beta-blockers first, and 8 of them (57%) specified the use of metoprolol. Only 2 centres (14%) prescribed sotalol as standard prophylaxis.

**Fig. 3:**
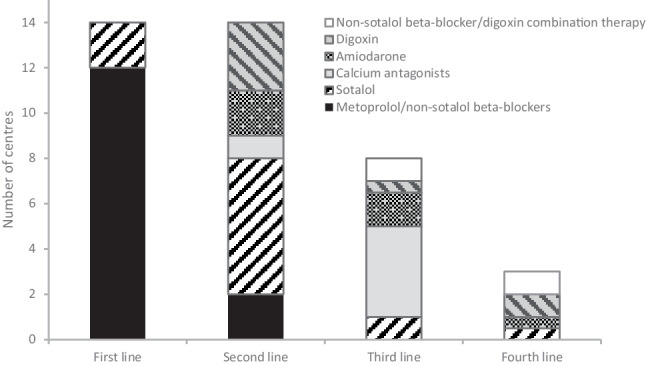
Reported prescription of postoperative medication for prevention and treatment of postoperative atrial fibrillation in Dutch cardiothoracic centres. Prophylaxis is first line of medication only

Digoxin, amiodarone and calcium antagonists were only used therapeutically when patients presented with POAF. All centres had a second line of medication, which comprised sotalol (43%), digoxin (21%), amiodarone (14%), non-sotalol beta-blockers (14%) and calcium antagonists (7%). Eight centres had a third line of treatment, which mostly consisted of a calcium antagonist (50%). One centre administered either amiodarone or digoxin as third-line medication. Three centres had a fourth line of treatment. One centre prescribed either amiodarone or sotalol as its fourth line. Furthermore, 2 centres prescribed combination therapy of non-sotalol beta-blockers and digoxin as its third- or fourth-line treatment. The reported first and subsequent lines of medication for the prevention and treatment of POAF are outlined in Fig. 3.

Overall, non-sotalol beta-blockers and sotalol took up 38 and 24%, respectively, of all medication prescribed postoperatively for POAF. Digoxin, calcium antagonists and amiodarone comprised 14, 13 and 10% of the prescriptions, respectively.

### Anticoagulant usage

All centres prescribed anticoagulants whilst treating POAF, but the indication differed. Six centres (43%) based their indication area once POAF occurred on national guidelines, 2 centres (14%) used the CHA_2_DS_2_-VASc score and national guidelines, another 2 centres only used the CHA_2_DS_2_-VASc score, 3 centres (21%) followed local protocols, and 1 centre used both its local protocol and the CHA_2_DS_2_-VASc score.

The question about the annual number of patients discharged with vitamin K antagonists (VKAs), was answered by 13 centres (93%): 4 (31%) replied this number was unknown and they could not provide a description of the trend, 7 (54%) reported an annual incidence of 0–56% (median 21%), and 2 (15%) could not provide an exact number but either stated a decrease in VKA prescriptions or an increase in direct oral anticoagulant (DOAC) prescriptions. The centre that did not prescribe VKAs to patients with POAF administered DOACs.

With regard to prescription duration, 1 centre was not aware how long its patients took VKAs. The majority (12/14; 86%) included the duration of VKA prescription in the referral letter to the Dutch Thrombosis Service. One centre prescribed anticoagulants indefinitely. None of the centres arranged a follow-up for their patients pertaining to VKA usage.

### Electrical cardioversion

**Fig. 4:**
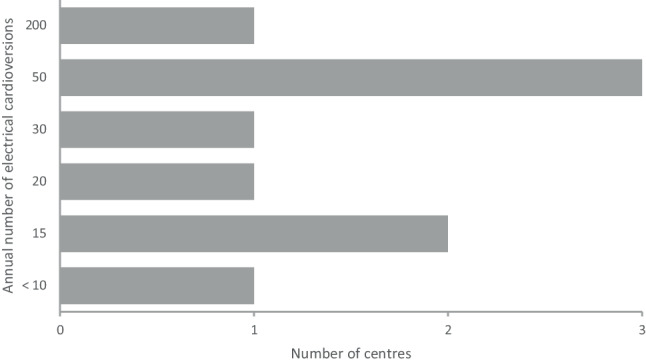
Reported number of electrical cardioversions performed yearly in Dutch cardiothoracic centres

Only 3 of the 14 centres (21%) did not perform standard ECV if chemical conversion failed, although 2 of them performed ECV on indication or in hemodynamically impaired patients. Twelve centres answered the question about the annual number of ECVs: 3 (25%) did not know the exact number and 9 (75%) provided an estimate, which ranged from < 10 to 200. Figure 4 shows the reported annual number of ECVs performed per centre.

### Perceived complications

**Fig. 5:**
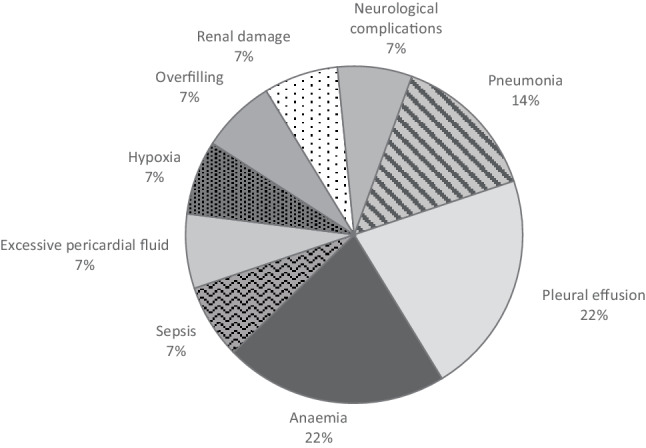
Perceived complications associated with postoperative atrial fibrillation in Dutch cardiothoracic centres

Thirteen respondents (93%) noticed longer hospitalisation for patients with POAF. Additionally, 6 centres (43%) replied these patients generally had other complications. Anaemia and pleural effusion each comprised 21% of the perceived complications, pneumonia made up 14%, and the incidence of sepsis, excessive pericardial fluid, hypoxia, overfilling, renal damage and neurological complications was 7% each. The perceived complications associated with POAF are outlined in Fig. 5.

Five respondents (36%) did not notice POAF occurred more often after specific procedures, but 9 (64%) stated they did. Of those that did, 5 centres reported observing POAF mainly after mitral or any valvular surgery, 1 centre noticed POAF after coronary bypass, and the remaining 3 saw POAF more frequently after complex procedures, longer perfusion and clamping duration, and impaired left ventricle function.

### Postoperative atrial fibrillation risk score

None of the centres used the POAF risk score [20]. However, when asked about their willingness to implement it, 7 (50%) were positive, 5 (36%) were negative, and 2 (14%) were indifferent. Reasons for unwillingness were insubstantial support, no effective prevention, and no added benefits either due to low POAF rates at their centre or because the centre administered prophylaxis to all patients. Of the 13 centres that further elaborated on their answer, 12 were willing to use a similar risk score if proven sufficiently effective. One centre was indifferent, as it considered all patients undergoing surgery as high-risk.

### Recommendation on improvements

Half of the respondents (7/14) answered the question on what could be an improvement for Dutch centres regarding POAF management. They mainly wanted better national agreements or a consensus on preferred treatment for more uniform management. Others expressed wanting better designed guidelines for anticoagulant prescription and increased guidance on duration, especially after referral to the Thrombosis Service.

## Discussion

In this survey study, the incidence of POAF reported by Dutch cardiothoracic centres was 20–30%. Despite the use of different preventive strategies, the reported incidences were similar. Respondents highlighted the need for more uniform treatment. The use of non-pharmaceutical preventive interventions was limited as respondents were awaiting results of upcoming trials. The majority observed longer in-hospital stay for patients with POAF and noticed POAF was accompanied by other complications, primarily anaemia, pleural effusion and pneumonia. Furthermore, centres embraced concomitant LAAC as a relevant stroke prevention measure in patients with pre-existing AF. Moreover, the discharge of patients taking VKAs may need better follow-up to prevent unnecessary prolongation of medication usage. A remarkable aspect of our study was the varying number of ECVs, suggesting that additional guidelines that stipulate the exact indications are needed. Considering the lack of evidence on optimal anticoagulant strategies for incident and transient AF, it is important to acknowledge the need for further research, which should provide clear guidance on the optimal use and duration of anticoagulants in these specific patient populations.

Preoperative prophylaxis was implemented in 6 centres, of which 3 solely prescribed preoperative prophylaxis for patients already on therapy. The other 3 centres prescribed preoperative prophylaxis to all patients. All 14 centres administered postoperative prophylaxis. Respondents noticed POAF occurred more often after valvular surgery. Valvular AF is a common indication for valve surgery [1]. Although a link cannot yet be established, POAF following valvular surgery may be more common, possibly due to pre-existent advanced atrial cardiomyopathy—and this requires further study.

Colchicine was not prescribed pre-emptively, which is remarkable given the potential benefits [9]. However, there was also mention of gastro-intestinal side effects [9], and use of this drug will therefore require fine-tuning of the specific dose needed. Amiodarone administration was limited among medical centres, comprising 10% of the postoperative medication prescriptions. Furthermore, amiodarone was only used for treating purposes and not as a preventive drug. Notably, amiodarone is only recommended unless beta-blockers are contra-indicated, according to several guidelines [5, 21, 22]. Therefore, beta-blockers may often not be contra-indicated in the clinical setting. The 2020 ESC/EACTS Guidelines suggest combining amiodarone and beta-blockers, as this has an increased effect on reducing POAF [4]. In light of the newly found results, amiodarone should be considered more as a means to prevent POAF. Amiodarone does carry risks that, again, necessitate finetuning [7]. A lower cumulative dose (< 3000 mg) may still be effective while avoiding adverse effects, according to the ESC/EACTS Guidelines [4].

Reasons for not using the POAF risk score were lack of perceived benefits and effective preventive measures. The latter implies that, even if the risk score is reliable, there are currently no consistent prevention methods, which essentially undermines the prominence of predicting POAF.

### Study limitations

Several limitations need consideration. Firstly, we may not have detected all cases of POAF due to, for example, different durations of telemetry monitoring per centre or transfer of patients before the occurrence of POAF, resulting in underdiagnosis.

Additionally, 1 centre did not answer the questionnaire, and not all centres that responded answered each question. Continuous reminders aimed to maximise response and completion rates could have resolved this issue. Moreover, 1 centre may have had registration bias, given they did not score preoperative AF until 2019. Respondents were not asked if electrolyte imbalances were routinely regulated; however, we can assume Dutch centres view this as a routine intervention that is proven effective [11, 23, 24].

### Recommendations

As aforementioned, although POAF is a short-term incident, it is still clinically relevant due to its association with long-term complications [1, 2, 25]. Prophylactic administration of amiodarone and colchicine is suggested for POAF prevention, as well as the use of posterior left pericardiotomy. Many other strategies can be assessed, such as active tube clearance, (bi)atrial pacing, and mapping and ablation of autonomic ganglia [7, 13, 26, 27]. Even though usage of such methods may be low in Dutch centres, it may prove valuable to obtain opinions on the matter via a future questionnaire. Another suggestion could be a nationwide/multicentre prospective implementation trial for non-pharmaceutical interventions, as this will facilitate the use of non-pharmacological prophylaxis and provide research results and direct experience with these interventions amongst practitioners.

It is unknown whether it is necessary to completely eliminate POAF, given its potential role as an indicator of underlying cardiovascular risks [3], and it is uncertain whether preventing POAF reduces the risk of long-term cardiovascular events. Rather than solely targeting the elimination of POAF itself, it may be more clinically relevant to focus on preventing long-term cardiovascular complications in patients with POAF. This could involve emphasising preventive measures and management of risk factors for future cardiovascular events. Furthermore, patients scheduled for elective cardiac surgery could be screened using speckle-tracking echocardiography, a diagnostic tool for disclosing atrial cardiomyopathy. Patients with this condition are at risk for developing cardiovascular incidents, which are now paradoxically associated with POAF [28].

## Conclusion

This study demonstrated limited use of preoperative POAF prophylaxis and non-pharmaceutical measures in Dutch cardiothoracic centres. POAF was associated with other complications, mainly pneumonia, pleural effusion and anaemia, and was perceived to occur more often after valvular surgery. There was consensus on the need for better national guidelines to come to a uniform approach to prevent POAF and the need for regulated management of anticoagulant use and increased guidance on prescription duration for patients. Adjustment of current protocols via an implementation trial was considered necessary to facilitate—and implement—all aspects needing change highlighted in this study.

Furthermore, it is essential to highlight the insight that a proportion of patients scheduled for cardiac surgery may already have a preclinical state of atrial cardiomyopathy. Its diagnosis could act as a marker for adverse outcomes and may be used to enhance preventive measures and risk management in these patients.

### Supplementary Information

**Table S1** Completion rates of online questionnaire per question
