# Supplementary material for: Interventions to prevent postoperative atrial fibrillation in Dutch cardiothoracic centres: a survey study
Source: Neth Heart J. 2024 Feb 15;32(4):173–81. doi: 10.1007/s12471-023-01849-1 (PMC10951169; doi:10.1007/s12471-023-01849-1)
Supplement: Supplementary file 1 — Table S1 Completion rates of online questionnaire per question [file 12471_2023_1849_MOESM1_ESM.docx]

**Table S1** Completion rates of online questionnaire per question^a^

|  | Question | Completion rate (%) |
| --- | --- | --- |
| 1 | How many cardiac procedures (i.e. sternotomy on pump) are performed annually at your center? | 92.9 |
| 2 | What is the percentage of postoperative AF (POAF)? | 92.9 |
| 3 | Does your center have a prevention protocol for POAF? | 100 |
| 4 | If so, what does this protocol consist of? | 64.3 |
| 5 | If not, what does your center use to prevent POAF? Do you use the POAF predictive score? (doi.org/10.1161/JAHA.113.000752)   - If so, what is the consequence? - If not, do you want to start using it? - If not, why? | 100 |
| 6 | What do you think of the suggestion to take preventive measures based on a risk score? | 92.9 |
| 7 | Do you prescribe any of the following medication or measures preoperatively? ☐ Beta-blockers (non-sotalol)  ☐ Sotalol  ☐ Ca antagonists  ☐ Other medication and/or measures, namely  Dosage: | 100 |
| 8 | Have you heard of Botox injections into the left atrial fat pad?   - Do you apply this procedure? - How do you feel about this procedure? | 100 |
| 9 | Have you heard of perioperative pericardial flush/rinsing?   - Do you apply this procedure? - How do you feel about this procedure? | 92.9 |
| 0 | Have you heard of posterior left pericardiotomy?   - Do you apply this procedure? - How do you feel about this measure? | 92.9 |
| 11 | Do you prescribe any of the following drugs postoperatively; in what order?  ☐Beta-blockers (non-sotalol)  ☐ Sotalol  ☐ Ca antagonists  ☐ Other medication, namely:  Dosage: | 100 |
| 12 | Is left atrial appendage closure (LAAC) performed concomitantly during heart surgery at your center? | 100 |
| 13 | Did you know there is a higher incidence of POAF following LAAC? | 100 |
| 14 | What is your indication area for LAAC? | 100 |
| 15 | Do you administer anticoagulants for POAF? | 100 |
| 16 | According to what protocol? | 100 |
| 17 | If chemical conversion cannot be achieved during admission, do you perform electrical cardioversion (ECV)? | 92.9 |
| 18 | Approximately how many ECVs do you perform on an annual basis? | 85.7 |
| 19 | Approximately how many patients do you discharge yearly with vitamin K/ thrombosis service medication? | 92.9 |
| 20 | How do you know when the referrer will stop this medication after SR returns? | 100 |
| 21 | What could be a relevant qualitative advancement in the Netherlands? | 50 |
| 22 | Do you have the impression patients with POAF are hospitalized longer? | 100 |
| 23 | Do you have the impression patients with POAF have more complications? | 100 |
| 24 | If so, what are examples of complications patients are more likely to have? | 57.1 |
| 25 | Do you feel POAF is more common after specific interventions? | 100 |
| 26 | If so, which interventions does this pertain to? | 57.1 |
| 27 | May we receive your protocol for POAF prevention? | 85.7 |
|  | **Total average** | **90.7** |

^a^ Questions have been abbreviated
